# A Proposed Waterpipe Emissions Topography Protocol Reflecting Natural Environment User Behaviour

**DOI:** 10.3390/ijerph17010092

**Published:** 2019-12-21

**Authors:** Edward C. Hensel, Samantha Emma Sarles, Abdulaziz al-Olayan, A. Gary DiFrancesco, Shehan Jayasekera, Nathan C. Eddingsaas, Risa J. Robinson

**Affiliations:** 1 Department of Mechanical Engineering, Rochester Institute of Technology, Rochester, NY 14623, USA; ses9066@rit.edu (S.E.S.); agdpci@rit.edu (A.G.D.); gbj6142@rit.edu (S.J.); rjreme@rit.edu (R.J.R.); 2 Department of Industrial and Systems Engineering, Rochester Institute of Technology, Rochester, NY 14623, USA; aaa4858@rit.edu; 3 Rochester Institute of Technology, School of Chemistry and Materials Science, Rochester, NY 14623, USA; ncesch@rit.edu

**Keywords:** nicotine, non-cigarette tobacco products, smoking topography, toxicology, public policy

## Abstract

Usage of waterpipes is growing in popularity around the world. Limited waterpipe natural environment topography data reduces the ability of the research community to accurately assess emissions and user exposure to toxicants. A portable ergonomic waterpipe monitor was provided to study participants to use every time they smoked their own waterpipe during a one-week monitoring period in conjunction with their own choice shisha tobacco. Users provided demographic information and logged their product use to supplement electronic monitor data. A total of 44 prospective study participants were invited to an intake appointment following an on-line pre-screening survey. Of these, 34 individuals were invited to participate in the study and data for 24 individuals who completed all aspects of the 1-week monitoring protocol is presented. 7493 puffs were observed during 74 waterpipe sessions accumulating over 48 h of waterpipe usage. The 95% CI on mean puff flow rate, duration, volume and interval are presented, yielding grand means of 243 [mL/s], 3.5 [s], 850 [mL], and 28 [s] respectively. The middle 95% of puff flow rates ranged between 62 to 408 [mL/s], durations from 0.8 to 6.8 [s], and puff volumes from 87 to 1762 [mL]. A waterpipe emissions topography protocol consisting of 13 flow conditions is proposed to reflect 93% of the observed range of puff flow rate, puff duration and puff volume with representative inter-puff interval, cumulative session time and aerosol volumes.

## 1. Introduction

The 2016 consensus statement by Maziak et al. [1] concluded that waterpipe smoking is emerging as a global threat to public health. There is a need to better understand the natural environment topography behaviour of waterpipe users in a variety of own-choice settings, such as in personal residences. Maziak et al. [1] presents four research focus areas and 33 core questions for assessment of waterpipe use. The paper highlights the importance of capturing variability in use patterns related to waterpipe smoking that exists due to the social aspect of the product and its set-up time. Information gathered from natural environment monitoring may inform protocols for emissions testing of waterpipe and shisha products. Such emissions tests may provide insight regarding the joint impact of user behaviour and product characteristics on total particulate mass and Hazardous and Potentially Hazardous Constituent (HPHC) exposure. This improved body of knowledge may inform the regulatory science community and enhance health guidance from clinicians to patients.

Perhaps the earliest field assessment of waterpipe topography was conducted in 2004 [2], in a café setting nearby the American University of Beirut, Lebanon, using an instrumented waterpipe. They observed the first 30 min of a typical 90-min session for 52 individual smokers and recorded flow rate versus time. The resulting mean topography parameters established the Beirut Protocol for laboratory smoking machine studies consisting of 171 puffs during a 61 min session, composed of individual puffs having a puff volume of 530 milliliters [mL], duration of 2.6 seconds [s] and inter-puff interval of 17 s, yielding a nominal mean puff flow rate of 204 mL/s (the average volumetric flow rate during a puff expressed in milliliters per second) and session volume of about 93 L (the summation of the volume of all puffs taken by a smoker during an entire waterpipe smoking session). Collectively these characteristics describing a smoker’s puffing behaviour are referred to as the ‘topography’ of the smoker. Topography parameters may be reported as discrete values for an individual puff, as descriptive statistics for all puffs taken by a single smoker (e.g., mean, standard deviation, confidence interval) or as descriptive statistics describing a cohort of smokers. Since then, there have been several waterpipe topography studies in the lab [3,4,5], natural environment café [2,6,7,8] and in home [6] as summarized in Jawad [6], reporting a wide range of topography parameters; 160 to 300 [mL/s] for flow rate, 2.4 to 3.9 s for puff duration, 440 to 906 mL for puff volume. Studies typically observed use over a 45-min period ranging from 30.7 min to 64.0 min [2,3,4,5,6,7,8].

There is wide variability in puffing behavior and toxicant inhalation expected in a natural setting, such that one mean topography puffing regime may not be sufficient to assess the range of potential risk from waterpipe use. Capturing the full puffing regime is critical for emissions testing; flow rate has a strong correlation to total particulate matter, including HPHC aerosol constituent exposure [9,10]. Therefore, a protocol which statistically covers the range of natural use behaviour is needed and such a protocol should be derived from data obtained within the natural use environment. This work aims to establish such a protocol.

## 2. Materials and Methods

### 2.1. Study Protocol

The observational study protocol employed in this study follows the same structure as with prior studies conducted by the research group, consisting of a 24-h cig-a-like study [11], a one-week own-choice e-cig study [12], and a two-week vape pen flavor-switching study [13]. The protocol consisted of the following phases. The study protocol was reviewed and approved by the Rochester Institute of Technology (RIT) Institutional Review Board (IRB).

#### 2.1.1. Recruitment

The recruitment communications population consisted of adult waterpipe users affiliated with the Rochester Institute of Technology (RIT) campus community which includes approximately 15,950 undergraduate students, 3100 graduate students, and 4000 faculty and staff. The student population includes approximately 1000 deaf and hard-of-hearing students. Participants were recruited via mass-emails sent to the campus distribution list in conjunction with flyers posted around the campus. The email and flyers advertised a research study regarding waterpipe usage and stated that participants may be eligible to receive $50 for participating in a 1-week study, if they were a current waterpipe user and over 18 years of age. Anyone interested was asked to contact the research administrator at the email provided.

#### 2.1.2. Pre-Screening

The research administrator responded to each email received from a potential participant with detailed information about the study and a link to a pre-screening questionnaire. The online pre-screening questionnaire identified and excluded individuals who did not meet the eligibility requirements. Individuals passed the pre-screening if their responses indicated that they consented to participate, were between 18–65 years old, and were current waterpipe users as defined by the standard PhenX Protocol PX741401, “Use of Tobacco Products” [14] and were invited to schedule an intake appointment at the Respiratory Technologies Lab (RTL) at RIT. The PhenX questionnaire was used to determine if respondents were users of a single product, such as waterpipe, dual users (i.e., cigarettes and waterpipe) or poly users (multiple forms of tobacco products). Individuals who did not pass the pre-screening were notified immediately after taking the survey.

#### 2.1.3. Pre-Deployment Topography Monitor Calibration

Prior to each participant’s intake appointment, a technician conducted a pre-deployment flow rate calibration of the wPUM™ waterpipe (Rochester Institute of Technology, Rochester, NY, USA) topography monitor [15] assigned to that participant. Flow rate calibration was done using the fully characterized RIT PES-2™ (Rochester Institute of Technology, Rochester, NY, USA) emissions and calibration system, which employs flow meters certified annually by a third-party vendor. Each calibration resulted in a calibration curve relating individual wPUM™ topography monitor raw voltage readings to the primary instrument flow rate measurement.

#### 2.1.4. Intake Appointment

Intake appointments took place in the RTL on Tuesday, Wednesday or Thursday of each week, so that full day weekend and weekday behavior could be captured. Each appointment lasted between fifteen minutes to one hour, and included final screening via interview, verification of age by government-issued identification, informed consent, and verbal confirmation of the participant’s waterpipe product use. All potential participants were asked if they wanted to quit smoking (use of waterpipe) and if so, were provided with cessation information. Potential participants were excluded if they had been diagnosed with any of the following: bronchiectasis, lung cancer, asthma with reversibility on post bronchodilator spirometry, COPD, cystic fibrosis, allergic bronchopulmonary aspergillosis, pneumonia in prior six weeks, systemic corticosteroids, history of other significant chronic lung disease, pregnancy or intent to become pregnant, congestive heart failure, other chronic systemic illness, smokers on antioxidants, or anti-inflammatory therapy, or recent (last two weeks) upper respiratory tract infection. Once enrolled, participants were provided with a wPUM™ topography monitor, carrying case and paper study-log and assigned a unique number. Participants were instructed on the proper use of the portable wPUM™ waterpipe topography monitor in conjunction with the participant’s own waterpipe (participants were not provided with a waterpipe) and given an opportunity to turn the monitor on and off in the lab. Participants were invited to contact the research administrator during the monitoring period if they encountered any difficulties.

#### 2.1.5. Monitoring Period

The monitoring period began immediately after the intake appointment and lasted for seven days to capture weekday and weekend behavior without interruption. Participants were instructed to smoke waterpipe naturally in their own environment using their own waterpipe and shisha tobacco. The wPUM™ waterpipe topography monitor, which acts as a replacement mouth pipe and hose, was to be used for every smoking session, whether at their home or elsewhere. Participants were invited to optionally share photographs of the monitor with their own choice waterpipe. Additionally, each participant was instructed to complete a daily study log. The study log provided details from each participant about the day of each smoking session, the brand and flavor of tobacco shisha used, presence of other non-monitored smokers, number and type of coals used, session duration and location. Each participant was provided with contact information for the research administrator and invited to contact them if any questions or concerns arose during the monitoring period.

#### 2.1.6. Outtake Appointment

Outtake appointments took place in the RTL. Participants returned the wPUM™ waterpipe topography monitor, carrying case and daily study log to the RTL. The research administrator conducted an exit interview to assess product and monitor use during the observation period, identify difficulties encountered during the study, and solicit features which might improve usability of the monitor.

#### 2.1.7. Post-Deployment Topography Monitor Calibration

Post-deployment calibrations were conducted on each wPUM™ waterpipe topography monitor to determine if the monitor had been damaged, affected by fluid or particulate build-up, or any other variation which might adversely impact monitor performance.

### 2.2. Materials and Equipment

**Figure 1:**
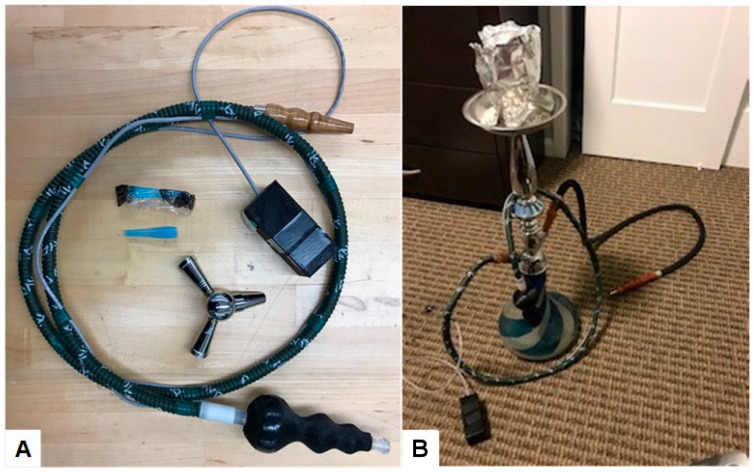
Panel (**A**): wPUM™ waterpipe monitor prepared for deployment. Panel (**B**): RIT wPUM™ waterpipe monitor ready for use in a participant’s natural environment with their own-choice personal waterpipe. Each participant is provided a kit consisting of a carrying case, y-splitter, disposable mouthpiece, and monitor with hose.

The wPUM™ waterpipe topography consisted of a mouth pipe or handle, hose, and electronics box, as illustrated in Figure 1, panel A. The proximal end of the monitor was modelled to present the same ergonomic hand grip, mouthpiece and flow diameter of a commercially available waterpipe hose. The distal end of the monitor consisted of a standard commercially available waterpipe hose and standard waterpipe interface. Each participant inserted the distal end of the monitor into the receiver of their own choice waterpipe during each smoking session over the course of the monitoring period. Other individuals could use a separately attached hose on the same waterpipe while the participant used the monitor hose. Panel B of Figure 1 shows a photograph, provided by a participant, of their own choice waterpipe used “at home” during study. This participant connected the monitor to the left port of their waterpipe and connected a second personal hose to the right port for another smoker. Users were provided with disposable mouthpieces for their use if desired, and a “y splitter” in case they chose to use their waterpipe with a non-consented smoker on a second hose. Every monitor used in this study exhibited a pre-post calibration repeatability of r_pre,post_ ≥ 0.972.

### 2.3. Data Analysis Procedures

Topography data analysis consists of four phases: (1) data integrity and quality review, (2) signal processing and puff identification, (3) descriptive statistics and behavior analysis, and (4) inferential statistics and comparative analysis.

Phase 1 (data integrity and quality review) consists of extracting raw voltage data from the monitor, comparing information against the study log for consistency and protocol compliance, assessment of the monitor pre/post repeatability, and concludes with a decision whether to exclude data from any participant due to (a) not completing all aspects of the one-week duration study protocol, (b) not complying with use of the waterpipe monitor, or (c) evidence of improper monitor use or monitor failure. All exclusions and reasons for such exclusions are recorded.

Phase 2 (signal processing and puff identification) consists of three discrete steps. Step 1 includes a low pass smoothing filter, checks for monitor fouling, and excludes exhale puffs. Step 2 converts the voltage data into instantaneous flow rate and employs a threshold of 45 [mL/s] flow rate (the instantaneous puff flow rate is recorded at the rate of 40 samples per second) to identify onset and conclusion of discrete puffs. The mean puff flow rate is computed as the arithmetic average of all flow rate samples recorded during a single puff. Step 3 consists of integrating the instantaneous puff flow rate across all puffs within a smoking session to estimate the cumulative session volume.

Phase 3 (descriptive statistics and behavior) analysis commences after the topography data for each session has concluded for each participant, to estimate the minimum, mean, maximum, standard deviation and confidence intervals on topography parameters (puff flow rate, duration, volume and interval) and consumption parameters (session, daily, and weekly puff time of day, puff count, and cumulative puff volume). ‘Topography’ parameters typically refer to “how” a smoker behaves during the act of taking a puff, while ‘consumption’ parameters refer to “how much” tobacco product a smoker consumes during a specified interval of time.

Phase 4 (inferential statistics and comparative) analysis combines the data from individual participants to assess inter-subject variation and group-wise topography characteristics.

## 3. Results

### 3.1. Study Cohort

**Figure 2:**
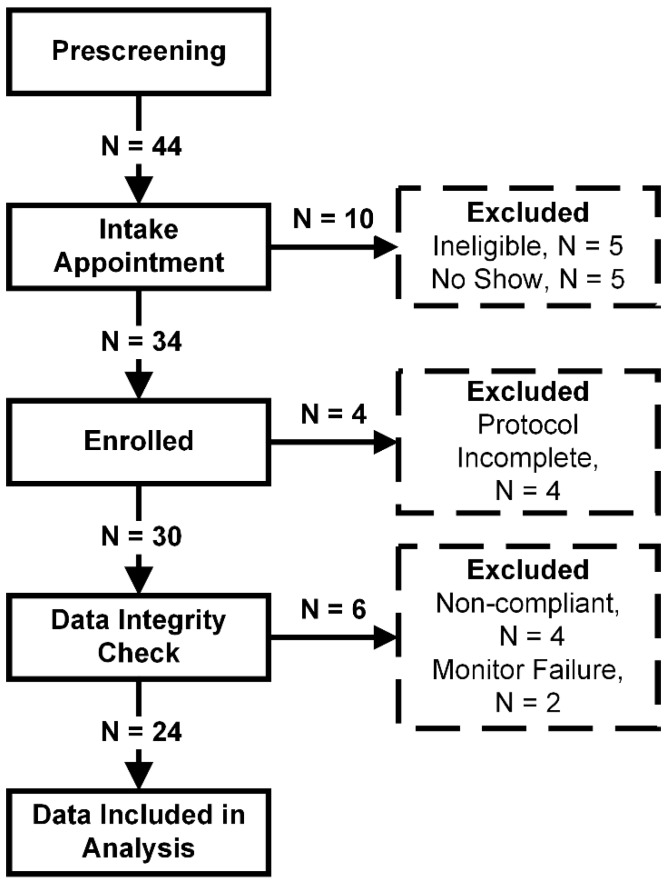
Cohort study flow chart. N = 44 participants were invited to attend an intake appointment, N = 34 respondents were found eligible, N = 34 participants were enrolled. Data from N = 4 participants were excluded from the analysis for data integrity reasons and N = 6 participants did not complete the observation protocol (one had an unrelated injury, three were not compliant, and two participants returned a broken monitor). Data from all remaining N = 24 participants is presented in the results.

The flow of participants through the study is illustrated in Figure 2. Of the 44 individuals who passed the pre-screening survey and were invited to schedule an intake appointment, five failed to attend their scheduled intake appointment, and five did not pass the eligibility criteria assessed at their intake interview. A total of 34 participants were thus enrolled and assigned a unique identifier from “1” to “34”. Of the 34 enrolled participants, four withdrew from the study prior to the exit interview for various personal reasons and 30 attended the exit interview.

During Phase 1 (data integrity and quality review), four participants were excluded due to their clear non-compliance with the study protocol (one due to an unrelated injury, two who falsely claimed usage during the exit interview but did not record any data, and one who claimed usage but the monitor recorded only exhale puffing of a nature completely inconsistent with waterpipe use). Each monitor was confirmed to be in proper working order during pre/post-calibration for the three participants who appeared to falsify their study participation. In addition, two participants were excluded due to monitor hardware failure (in this case, both participants 3 and 4, roommates, used the same monitor during shared sessions). The data from the remaining 24 participants were included in the analyses presented here. While a Y-splitter was provided all study participants, no participants reported use of the Y-splitter on their study logs or in the exit interview.

The final study cohort consisted of four females and 19 males with an average reported age of 24 (3.2 STD) years. Supplement Table S1 summarizes the detailed demographics, interview results and usage log summary reported by the 24 participants whose data were included in the analyses.

**Figure 3:**
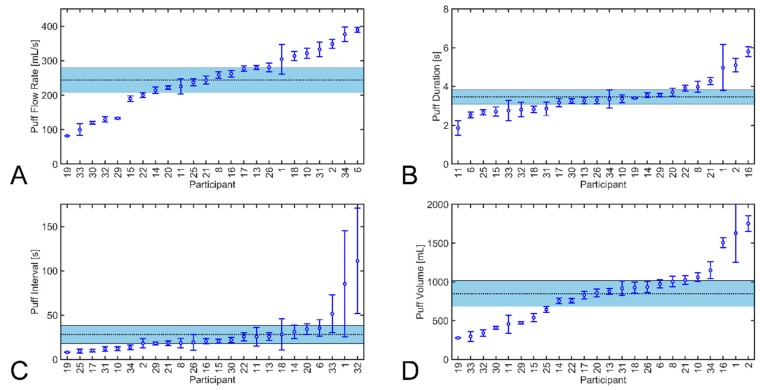
Topography characteristics of participants, sorted from low to high, are shown as mean value with 95% confidence intervals on each quantity: Panel (**A**) mean puff flow rate, Panel (**B**) puff duration, Panel (**C**) puff interval, and Panel (**D**) puff volume. The shaded area indicates the group-wise grand mean and 95% confidence interval of each quantity.

Every participant reported using their personal waterpipe for most smoking sessions in their own residence. Most waterpipe sessions were reported as single-user sessions, with 5 of the 24 participants reported participating in multi-user sessions. Participants 1 and 2 were roommates and shared one monitor. Participant 2 smoked a single session sharing the monitor with Participant 1 (denoted ‘2+1’ in Figure 3) and Participant 1 smoked one additional session alone. Participants 8, 10 and 11 had multi-user sessions wherein the other user(s) were not monitored. Participants reported use of both natural and instant light charcoal in conjunction with a variety of shisha flavors and brands, as detailed in the supplemental material. The most commonly reported shisha consumed was “Two Apple” flavor by Al Fakher^®^ (Al Fakher Tobacco Trading, P.O. Box 20037, Ajman, United Arab Emirates) purchased individually by study participants. 

### 3.2. Topography Behavior

The mean topography characteristics (puff flow rate, duration, volume and inter-puff interval) for 24 participants are shown in Figure 3, along with grand mean and 95% confidence intervals for each quantity. The data in each plot is sorted from low to high mean value.

Supplement Table S2 summarizes, for each participant, the total puff count and number of sessions collected with the monitor. Additional topography details are provided, including mean session duration and cumulative aerosol volume and standard deviations thereof. The average compliance of monitor usage for 24 participants was 97% based on comparing topography session count to self-reported session count. Individual participant mean puff flow rate ranged from 82 to 389 mL/s with a group mean of 243 mL/s, puff duration from 1.9 to 5.8 s with a group mean of 3.5 s, puff volume from 278 to 1751 mL with a group mean of 850 mL, and inter-puff interval ranged from 8 to 111 s with a group mean of 28 s. The mean cumulative session aerosol volume ranged from 6 L to 231 L with a group mean of 77 L. The individual participant mean session duration ranged from 17 to 61 [min] with a group mean of 41 min.

**Figure 4:**
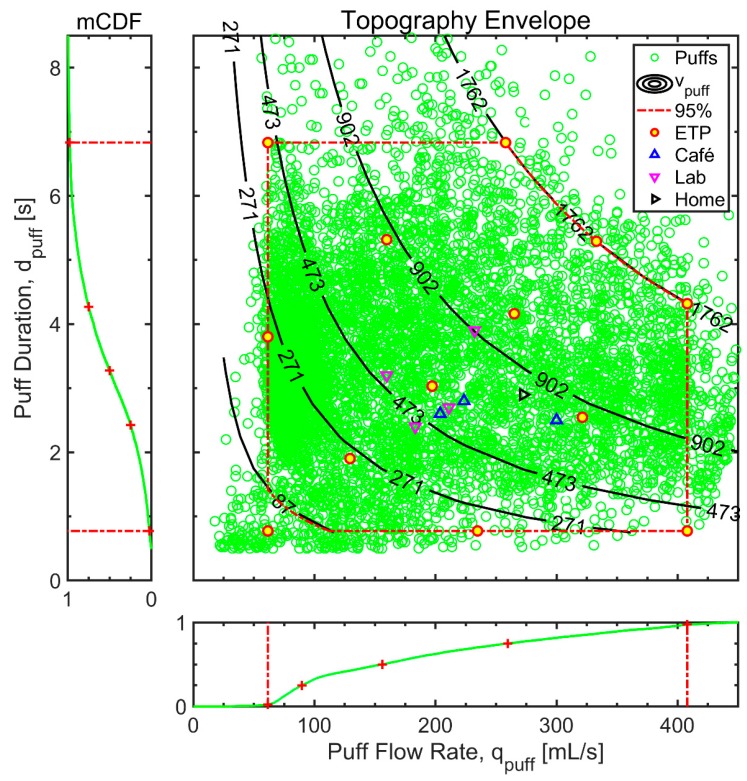
The discrete puff topography data are presented as a function of puff flow rate and duration, with lines of constant puff volume shown as contours. Individual data points represent each discrete puff taken by 24 waterpipe users during a one-week natural environment monitoring period. The empirical marginal CDFs as a function of puff flow rate and puff duration are shown along the horizontal and vertical axes respectively. A topography envelope indicating approximately 93% of all puffs observed and 13 conditions proposed for the waterpipe emissions topography protocol (ETP) flow conditions (see Discussion and Table 1) are super-imposed on the data along with topography data reported in the literature from clinical laboratory [3,4,5], in home [6], and café settings [2,6,7,8].

The discrete puff data from all 24 participants is presented in Figure 4, to show the prevalence of topography behavior as a function of puff flow rate, duration and volume. The data represents N = 7493 individual puffs taken and was analyzed to determine the empirical probability density function (PDF), which was then integrated to estimate the joint cumulative distribution function (CDF) of the study cohort. The marginal CDF of puff flow rate is along the horizontal axis while the marginal CDF of puff duration is presented along the vertical.

The marginal Cumulative Distribution Function, mCDF, of puff duration indicates that 95% (0.025 < mCDF(d) < 0.975) of the puffs have puff durations between approximately 0.8 and 6.8 s. The marginal CDF of puff flow rate indicates that 95% (0.025 < mCDF(q) < 0.975) of the puff flow rates range between approximately 62 to 408 mL/s. Similarly, the middle 95% of puff volumes range from approximately 87 to 1762 mL. We use these bounds to define the “topography envelope” which spans the range of waterpipe use topography observed by smokers in their natural environment. The topography envelope, illustrated by the dash-dot red line bounding box in Figure 4, informs the range of flow conditions needed to fully characterize waterpipe use in residential environments. The 25th, 50th, and 75th percentile of puff duration and mean puff flow rate are indicated by the red ‘+’ markers on the (2.4, 3.3, 4.3 s) left and (90, 156, 260 mL/s) lower panels, respectively. The 25th, 50th, and 75th percentile of puff volume are illustrated by iso-volume contours (271, 473, 902 mL) on the main panel. We note that 93% of the 7493 puffs observed lie within the topography envelope.

## 4. Discussion

The current study of 24 young adults using personally owned waterpipes in a residential setting, primarily in their own residence, demonstrated substantial inter-subject variability with respect to topography parameters. Waterpipe topography data reported previously [2,3,4,5,6,7,8] for a café environment (upward triangles on Figure 4) and a clinical laboratory environment (downward triangles on Figure 4), are consistent with data reported herein. Our data suggests a significantly expanded range of topographies is required to fully characterize emissions from waterpipe which reflect the range of observed use behaviour. There is wide intra-subject and inter-subject variation in the topography among waterpipe users, which may have a significant effect on the emissions and hence the toxicant exposure of individuals. A single representative machine puffing profile is insufficient to characterize waterpipe emissions and exposure to HPHCs.

We propose the family of waterpipe Emissions Topography Protocol (ETP) flow conditions shown in Table 1 be used to fully characterize the emissions profile of waterpipes across the range of topography behaviors exhibited by users in their natural environment. The proposed ETP flow conditions span the range of all prior reported studies of waterpipe use in café, residential, and laboratory settings, and is illustrated by the thick border, filled circles in Figure 4.

The proposed ETP consists of 13 topographies spanning 95% of the puff flow rate range, 95% of the puff duration range, and 95% of the puff duration range. The ETP is proposed as a means for the research community to collectively characterize emissions from a variety of waterpipes and consumables across the range of topography behavior. The pattern is chosen to permit subsequent linear triangular interpolation of laboratory emissions test results between ETP. The ETP is proposed as a means to fully characterize a waterpipe device (the waterpipe itself) for a given configuration (e.g., number of coals) and a given tobacco product (shisha nicotine concentration, flavor or sugar additives, propylene glycol to glycerin ratios, other humectants, etc.). As the research community builds a library of device and consumable product characterizations in accordance with the ETP, we can better understand the effect of proposed regulatory changes (such as shisha composition) on emissions. The 14 flow conditions presented in Table 1 may be conducted in any convenient experimental order. The inter-puff interval was chosen to be constant at 25 s to be representative of user behavior in the natural environment. The interval was maintained constant between flow conditions to provide sufficient time between puffs so the experimentalist can periodically swap out filter pads, in the emissions collection system, typically after every twenty puffs. Experimental results from a reduced set of ten flow conditions has been reported previously [9,10] to illustrate how the total particulate matter and toxicant yields (such as aldehydes) may be reported on a mass concentration, mass ratio, and time-dependent basis. Prior work demonstrated that flow conditions impact the emissions. In order to realistically conduct an accurate comparative risk assessment of toxicants from two waterpipe tobacco compositions (perhaps one with low humectant content and another with high humectant content) the emissions should be analyzed over the entire range of realistic flow conditions. For example, the mean puff flow rate was previously shown to affect the coal and waterpipe body temperature which could impact aldehyde yields. If the product comparison is conducted under a single flow condition, then significant differences in the toxicant yield between the products may be overlooked. The session duration of 45 min was chosen as a fixed duration in an effort to make experimental results directly comparable between flow conditions, and also reflecting the reasonable combustion duration for a session using a single coal. A study which investigates the impact of coal mass on emissions may require a longer session to capture the entire combustion period of the coal.

Expanding upon a framework proposed previously for e-cigs [16,17], we project that personalized estimates of toxicant yield (total particulate matter, nicotine, volatiles, etc.) delivered to the mouth of a user may be estimated from observations of their personal topography in conjunction with waterpipe emissions captured at the ETP for their chosen device and shisha. Experimental validation of this approach is ongoing [9,10] and will be presented in a future work. Combination of the emissions topography protocol with experimental data collection and the mathematical framework may eventually lead to enhanced predictive modeling of relationships between product characteristics and aerosol emissions.

The topography envelope presented herein was collected from 24 users during a single week of observation in a single geographic location and across a limited set of waterpipes and chosen waterpipe tobacco products. As additional researchers collect and report natural environment topography puff-by-puff for other settings and locations, a comprehensive quantitative understanding of the waterpipe topography envelope can be realized by the research community. Further, as the emissions characteristics of waterpipe and consumable are better understood as a function of the proposed ETP, the flow conditions can be refined to focus on areas of highest gradient in emission change as a function of topography, or the number density of test conditions can be increased to refine the fidelity of the time-dependent emissions surface.

## 5. Conclusions

In conclusion, this work presents the natural environment topography behaviour of adult waterpipe users for one week in conjunction with their own choice waterpipe and shisha tobacco. Descriptive statistics of topography data for 24 young adults are used to document a representative range of puff flow rate and duration for residential waterpipe use. A set of flow conditions is proposed for emissions testing of various waterpipe products to permit well informed product regulations and lay the foundation for future personalized toxicant yield estimates. The work documents the intra-subject and inter-subject variability in waterpipe puffing topography which may be expected in the residential setting and demonstrates that comprehensive emissions analysis of residential waterpipe use requires evaluation of a range of flow conditions to accurately reflect the usage behaviour.

## Figures and Tables

**Table 1:** Proposed waterpipe emissions topography protocol flow conditions. The recommended protocol consists of 13 conditions to reflect approximately 93% of the observed waterpipe puff flow rate, puff duration, and puff volume user behaviour with representative inter-puff interval, cumulative session time and aerosol volumes.

Condition Number	Puff Count	Puff Flow Rate	Puff Duration	Puff Volume	Inter-Puff Interval	Session Duration	Session Volume
[-]	[-/session]	[mL/s]	[s]	[mL]	[s]	[min]	[mL/session]
1	105	62	0.8	50	25	45.2	5191
2	105	408	0.8	326	25	45.2	34,158
3	85	62	6.8	422	25	45.1	35,796
4	92	408	4.3	1754	25	45.1	161,668
5	85	258	6.8	1754	25	45.1	148,958
6	89	333	5.3	1765	25	44.9	157,268
7	100	129	1.9	245	25	45.1	24,601
8	96	197	3.0	591	25	44.9	56,989
9	92	265	4.2	1113	25	44.8	102,914
10	105	235	0.8	188	25	45.2	19,674
11	94	62	3.8	236	25	45.1	22,088
12	98	321	2.5	803	25	45.0	78,791
13	89	160	5.3	848	25	44.9	75,564

## Supplementary Materials

The following are available online at https://www.mdpi.com/1660-4601/17/1/92/s1, Table S1: Self-reported participant demographic and product usage information. Table S2: Puffing topography observed of 24 waterpipe users.
